# Supplementary material for: Human papillomavirus genotype and cycle threshold value from self-samples and risk of high-grade cervical lesions: A post hoc analysis of a modified stepped-wedge implementation feasibility trial
Source: PLoS Med. 2024 Dec 12;21(12):e1004494. doi: 10.1371/journal.pmed.1004494 (PMC11637256; doi:10.1371/journal.pmed.1004494)
Supplement: S1 Table — (DOCX) [file pmed.1004494.s001.docx]

**Table S1 Baseline characteristics of study sample.**

| **Characteristics** | **N = 855** | **%^a^** |
| --- | --- | --- |
| **Age** |  |  |
| 25-29 years | 266 | 31.1% |
| 30-39 years | 326 | 38.1% |
| 40-49 years | 147 | 17.2% |
| 50-59 years | 78 | 9.1% |
| 60+ years | 38 | 4.4% |
| **Screening status^b^** |  |  |
| Not ≥6 m overdue | 13 | 1.5% |
| Late (≥6–<24 m) | 439 | 51.3% |
| Very late (≥24 m) | 167 | 19.6% |
| Never screened | 235 | 27.5% |
| Missing | 1 |  |
| **Ethnic background** |  |  |
| White | 313 | 48.6% |
| Asian | 110 | 17.1% |
| Black | 73 | 11.2% |
| Mixed | 116 | 18.0% |
| Other | 32 | 5.0% |
| Missing | 211 |  |
| **IMD (quintile)^c^** |  |  |
| Q1 (Most deprived) | 151 | 17.7% |
| Q2 | 361 | 42.3% |
| Q3 | 174 | 20.4% |
| Q4 | 112 | 13.1% |
| Q5 (most affluent) | 56 | 6.5% |
| Not available | 1 |  |

^a^percentega calculated only based on individual with non-missing values.

^b^Screening status defined as ‘Not ≥6 m overdue’ = screened within the last 3.5 years if aged 25–49 years or 5.5 years if aged 50–64 years , Late’ ≥6–<24 m overdue, ‘Very late’ = at least 24 m overdue; ‘Never’ = not screened previously (women aged <28 y without previous screens categorised as ‘late’).

^c^IMD, Index of multiple deprivation English Indices of Deprivation (ID) data for London at LSOA and borough level for the (ID2019) release. <https://data.london.gov.uk/dataset/indices-of-deprivation>.
