# Supplementary material for: Human papillomavirus genotype and cycle threshold value from self-samples and risk of high-grade cervical lesions: A post hoc analysis of a modified stepped-wedge implementation feasibility trial
Source: PLoS Med. 2024 Dec 12;21(12):e1004494. doi: 10.1371/journal.pmed.1004494 (PMC11637256; doi:10.1371/journal.pmed.1004494)
Supplement: S2 Table — (DOCX) [file pmed.1004494.s002.docx]

**Table S2 Proportion of HPV positivity in clinician-collected samples, referral to colposcopy, CIN2+ and CIN3+, by risk groups and age.**

| **Age and risk groups** |  | **hrHPV+ in CC samples** | | **Referred to coposcopy** | | **CIN2+** | | **CIN3+** | |
| --- | --- | --- | --- | --- | --- | --- | --- | --- | --- |
|  | N=855 | N | % | N | % | N | % | N | % |
| High risk |  |  |  |  |  |  |  |  |  |
| Age <30 years | 12 | 11 | 91.7% | 9 | 75.0% | 5 | 41.7% | 4 | 33.3% |
| Age 30-49 years | 29 | 27 | 93.1% | 24 | 82.8% | 13 | 44.8% | 10 | 34.5% |
| Age 50+ years | 3 | 3 | 100.0% | 3 | 100.0% | 1 | 33.3% | 0 | 0.0% |
| *all ages* | *44* | *41* | *93.2%* | *36* | *81.8%* | *19* | *43.2%* | *14* | *31.8%* |
| Intermediate risk | |  |  |  |  |  |  |  |  |
| Age <30 years | 116 | 85 | 73.3% | 55 | 47.4% | 13 | 11.2% | 4 | 3.4% |
| Age 30-49 years | 201 | 127 | 63.2% | 75 | 37.3% | 20 | 10.0% | 6 | 3.0% |
| Age 50+ years | 42 | 14 | 33.3% | 10 | 23.8% | 1 | 2.4% | 1 | 2.4% |
| *all ages* | *359* | *226* | *63.0%* | *140* | *39.0%* | *34* | *9.5%* | *11* | *3.1%* |
| Low risk |  |  |  |  |  |  |  |  |  |
| Age <30 years | 138 | 52 | 37.7% | 25 | 18.1% | 6 | 4.3% | 3 | 2.2% |
| Age 30-49 years | 243 | 81 | 33.3% | 40 | 16.5% | 10 | 4.1% | 5 | 2.1% |
| Age 50+ years | 71 | 21 | 29.6% | 8 | 11.3% | 2 | 2.8% | 1 | 1.4% |
| *all ages* | *452* | *154* | *34.1%* | *73* | *16.2%* | *18* | *4.0%* | *9* | *2.0%* |
| **Total** | **855** | **421** | **49.2%** | **249** | **29.1%** | **71** | **8.3%** | **34** | **4.0%** |

HPV, human papillomavirus; hrHPV, high-risk human papillomavirus; Ct, cycle threshold; CC: clinician-collected; CIN2+, cervaical intraepitheilial neoplasia grade 2 or worse; CIN3+, cervaical intraepitheilial neoplasia grade 3 or worse.

1. Only included women who had adequate cytology
2. Only women who had abnormal cytology were referred to colposcopy
