# Supplementary material for: Human papillomavirus genotype and cycle threshold value from self-samples and risk of high-grade cervical lesions: A post hoc analysis of a modified stepped-wedge implementation feasibility trial
Source: PLoS Med. 2024 Dec 12;21(12):e1004494. doi: 10.1371/journal.pmed.1004494 (PMC11637256; doi:10.1371/journal.pmed.1004494)
Supplement: S1 Fig — The predicted probabilities were generated by running mean smoothing (running line), logistic regression model with natural cubic spline (mkspline), natural cubic spline (spline), and fractional polynomial regression (fp); 95% CIs are illustrated for fractional polynomial regression (95% CI fp) and logistic regression model (95% CI mkspline). HPV, human papillomavirus; CI, confidence interval; CIN2+, cervical intraepithelial neoplasia grade 2 or worse. (DOCX) [file pmed.1004494.s003.docx]

**S1 Fig. Risk of CIN2+ and HPV Ct value.** The predicted probabilities were generated by running mean smoothing (running line), logistic regression model with natural cubic spline (mkspline), natural cubic spline (spline) and fractional polynomial regression (fp). 95% CIs are illustrated for fractional polynomial regression (95% CI fp) and logistic regression model (95% CI mkspline).

**
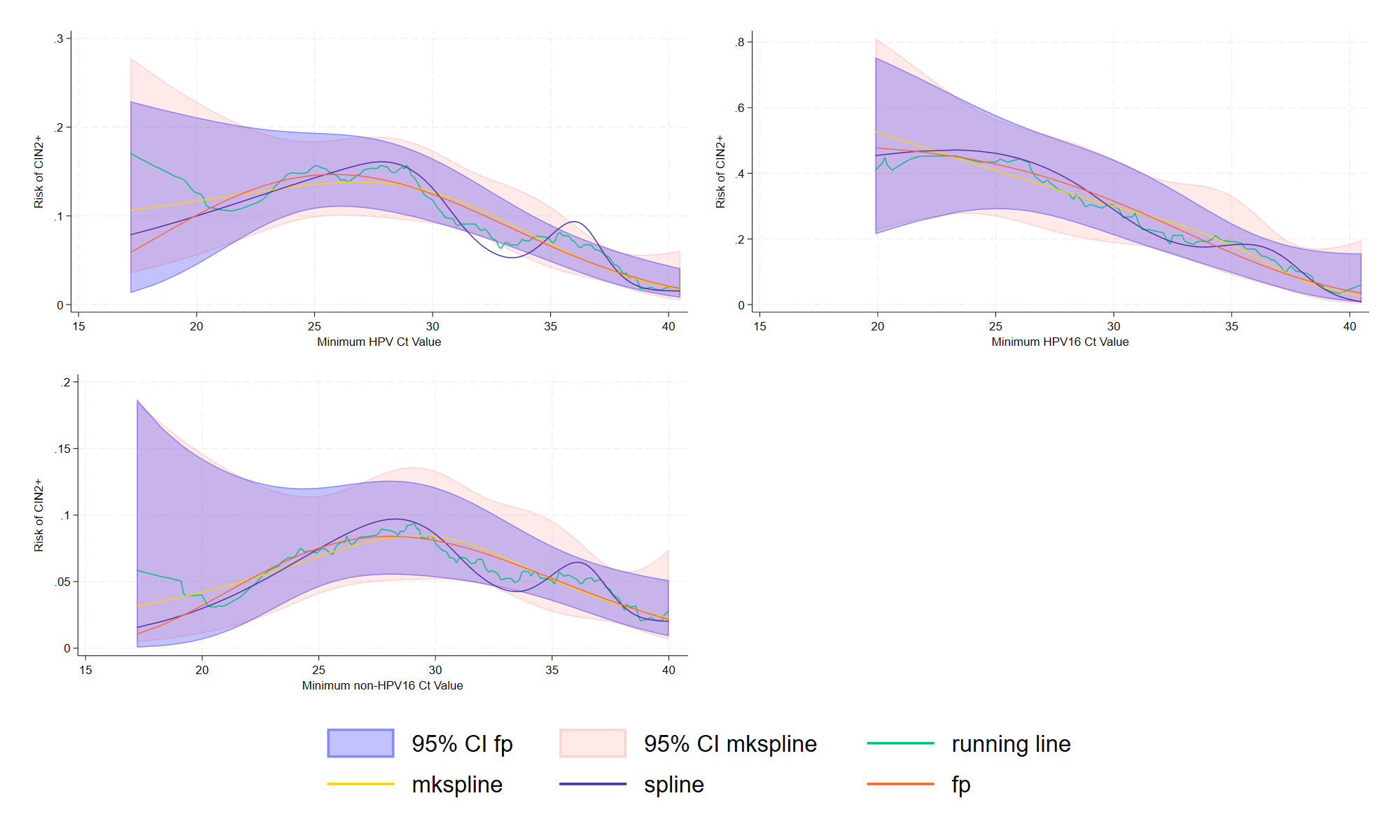
**

HPV, human papillomavirus; CI: confidence interval; CIN2+, cervical intraepithelial neoplasia grade 2 or worse
