# Supplementary material for: Human papillomavirus genotype and cycle threshold value from self-samples and risk of high-grade cervical lesions: A post hoc analysis of a modified stepped-wedge implementation feasibility trial
Source: PLoS Med. 2024 Dec 12;21(12):e1004494. doi: 10.1371/journal.pmed.1004494 (PMC11637256; doi:10.1371/journal.pmed.1004494)
Supplement: Attachment — Submitted filename: Ct paper General editorial requests.docx [file pmed.1004494.s008.docx]

**General editorial requests:**

(Note: not all will apply to your paper, but please check each item carefully)

*	We ask every co-author listed on the manuscript to fill in a contributing author statement, making sure to declare all competing interests. If any of the co-authors have not filled in the statement, we will remind them to do so when the paper is revised.

I believe that all authors have declared all competing interests. Please let us know if you are missing any.

*	Please upload any figures associated with your paper as individual TIF or EPS files with 300dpi resolution at resubmission; please read our figure guidelines for more information on our requirements:

We have uploaded TIF files of all Figures.

While revising your submission, please upload your figure files to the PACE digital diagnostic tool.

We used the PACE tool to check our Figures.

*	Please ensure that the paper adheres to the PLOS Data Availability Policy, which requires that all data underlying the study's findings be provided in a repository or as Supporting Information.

We are happy to provide data as Supporting information. We have not done so yet because (i) we are still discussing IP; and (ii) we want to ensure that the data will not enable identification of any participant. We plan to provide a spreadsheet with the following variables: age (5-year band), screening status (4 categories), ethnicity (5 categories), SES (5 levels of IMD), Ct beta-globin, Ct HPV16, Ct HPV18, Ct HPV other, clinical HPV samples result (positive/ negative), LBC result (not done, normal, abnormal), CIN2, CIN3+.

*	We expect all researchers with submissions to PLOS in which author-generated code underpins the findings in the manuscript to make all author-generated code available without restrictions upon publication of the work. In cases where code is central to the manuscript, we may require the code to be made available as a condition of publication. Authors are responsible for ensuring that the code is reusable and well documented. Please make any custom code available, either as part of your data deposition or as a supplementary file. Please add a sentence to your data availability statement regarding any code used in the study, e.g. "The code used in the analysis is available from Github [URL] and archived in Zenodo [DOI link]" Please review our guidelines and ensure that your code is shared in a way that follows best practice and facilitates reproducibility and reuse. Because Github depositions can be readily changed or deleted, we encourage you to make a permanent DOI'd copy (e.g. in Zenodo) and provide the URL.

We would be happy to add a Stata do-file to the Supplementary material.

FORMATTING - GENERAL

*	Abstract: Please structure your abstract using the PLOS Medicine headings (Background, Methods and Findings, Conclusions). Please combine the Methods and Findings sections into one section.

Done

*	At this stage, we ask that you include a short, non-technical Author Summary of your research to make findings accessible to a wide audience that includes both scientists and non-scientists. The Author Summary should immediately follow the Abstract in your revised manuscript. This text is subject to editorial change and should be distinct from the scientific abstract. Ideally each sub-heading should contain 2-3 single sentence, concise bullet points containing the most salient points from your study. In the final bullet point of 'What Do These Findings Mean?', please include the main limitations of the study in non-technical language.

We have added an Author Summary. We have used the recommended sub-headings.

*	Please express the main results with 95% CIs as well as p values. When reporting p values please report as p<0.001 and where higher as the exact p value p=0.002, for example. Throughout, suggest reporting statistical information as follows to improve clarity for the reader "22% (95% CI [13%,28%]; p</=)". Please be sure to define all numerical values at first use.

The main results are all presented with 95% confidence intervals. We have not included p-values because we are not focused on hypothesis testing.

*	Please include page numbers and line numbers in the manuscript file. Use continuous line numbers (do not restart the numbering on each page).

Done

*	Please cite the reference numbers in square brackets. Citations should precede punctuation.

Done

FIGURES AND TABLES

*	Please provide titles and legends for all figures and tables (including those in Supporting Information files).

Done

*	Please define all abbreviations used in each figure/table (including those in Supporting Information files).

Done

*	Please consider avoiding the use of red and green in order to make your figure more accessible to those with color blindness.

None of the Figures in the main paper include green. The supplementary Figure includes both red and green and we could redraw if required, but the important message from the Figure is that all the different curves are similar – it is not essential to identify which is which.

SUPPLEMENTARY MATERIAL

*	Please note that supplementary material will be posted as supplied by the authors. Therefore, please amend it according to the relevant comments outlined here.

Done

*	Please cite your Supporting Information as outlined here:

We made these changes to the way we cite Supporting Information

REFERENCES

*	PLOS uses the numbered citation (citation-sequence) method and first six authors, et al.

Done

*	Please ensure that journal name abbreviations match those found in the National Center for Biotechnology Information (NCBI) databases and are appropriately formatted and capitalised.

I believed we have

*	Where website addresses are cited, please include the complete URL and specify the date of access (e.g. [accessed: 12/06/2024]).

We wrote “[cited 10 May 2024]” if required we can change to “[accessed 10/05/2024]” (or even “05/10/224” if you prefer MM/DD/YYYY to DD/MM/YYY).

*	Please also see for further details on reference formatting.

Thank you

STUDY TYPE-SPECIFIC REQUESTS

*	Please be explicit in the title and the abstract that is a post-hoc sub-study/follow-on study of the primary trial. We suggest reporting in-line with CONSORT explicitly stating the sub-study nature and ensuring that the abstract details the main trial items in 2-3 sentences, including the study population, dates, intervention and primary outcome. The majority of the abstract should then describe the complete details of this post-hoc sub-study.

We have updated the title and the abstract to include “a post-hoc analysis”. We have added a couple of extra sentences to the Abstract describing the main trial. We have not included dates in the Abstract but we have in the Material & Methods section.

*	Please complete the CONSORT checklist and ensure that all components of CONSORT are present in the manuscript as well as clearly defined details of this sub-study. When completing the checklist, please use section and paragraph numbers, rather than page numbers as these often change in the event of publication.

We completed a CONSORT checklist for the main trial publication (in eClinicalMedicine). The checklist is for information to include when reporting a randomised trial. Since we are only reporting on a subset of individuals from the intervention arm in this paper, we did not think this was necessary. Much of the required information is already in the main publication and is not repeated here. Please let us know if you still require us to complete a CONSORT checklist and if so whether it is okay to cite where in the main paper the information can be found.

*	Please ensure that study registration details are included in the Methods section.

We have cited the registration ISRCTN:12759467 in the Methods section

*	Abstract: Please include the study design, population and setting, number of participants, years during which the study took place (original enrollment and follow up), length of follow up, and main outcome measures.

We believe that we have appropriately described the design of this “post-hoc study”. We did add this information for the RCT too, but I removed it just before resubmitting because it seemed irrelevant. This post-hoc analysis of a subset of participants who are all from the intervention arm does not report the main outcomes. They are already published. Please let us know if we must add these items of the original RCT design.

*	Please include absolute numbers wherever you report percentages; eg, n/N (%)

We have for point estimates (but not when the percentages are the limits of a confidence interval).

*	In keeping with our commitment to Open Science, please include the study protocol document and analysis plan (including any amendments) as Supporting Information to be published with the manuscript if accepted.

The main trial protocol is available via the ISRCTN website: <https://www.isrctn.com/ISRCTN12759467?q=12759467.&filters=&sort=&offset=1&totalResults=1&page=1&pageSize=10> Should we include it again in the Supplement or is it sufficient to give a link to the document? This analysis was not part of the original statistical analysis plan. There was no formal analysis plan for this post-hoc study. We could, if you would like, collate the e-mails in which I described the analyses that I wanted to Dr Lei to do. What do you think is appropriate?
