# Supplementary material for: Human papillomavirus genotype and cycle threshold value from self-samples and risk of high-grade cervical lesions: A post hoc analysis of a modified stepped-wedge implementation feasibility trial
Source: PLoS Med. 2024 Dec 12;21(12):e1004494. doi: 10.1371/journal.pmed.1004494 (PMC11637256; doi:10.1371/journal.pmed.1004494)
Supplement: Attachment — Submitted filename: Rebuttal_Letter_20241002.docx [file pmed.1004494.s009.docx]

**Response to comments on Manuscript (PMEDICINE-D-24-02470R2) entitled "** **Human papillomavirus genotype and cycle threshold value from self-samples and risk of high-grade cervical lesions: a post-hoc analysis of a modified stepped-wedge implementation feasibility trial"**

*GENERAL COMMENTS*

*1) When presenting age, please add a unit, such as 'years'. Please revise throughout the manuscript, including tables and figures (including those in the Supporting Information).*

Revised.

*2) Please check whether the details provided on lines 658-686 match the information provided in the online submission form.*

Checked.

*3) Thank you for your response regarding the CONSORT checklist. We will not be requiring you to complete the checklist,*

Thank you.

*4) There is no need to include the main trial protocol as a Supporting Information file, and since there was no formal study protocol document for the post hoc analysis, you do not need to include any document.*

Thank you.

*DATA AVAILABILTY*

*Please note that the data availability statement as written on lines 659-663 does not meet our journal requirements and does not match the statement provided in the online submission form. I have noted your comments in the point-by-point response and appreciate your efforts to provide the underlying data. Before resubmitting, please ensure that the paper complies with the PLOS Data Availability Policy.*

*For each data source used in your study:*

*a) If the data are freely or publicly available, note this and state the location of the data: within the paper, in Supporting Information files, or in a public repository (include the DOI or accession number).*

We have provided the data as supporting information file.

*b) If the data are owned by a third party but freely available upon request, please note this and state the owner of the data set and contact information for data requests (web or email address). Note that a study author cannot be the contact person for the data.*

*c) If the data are not freely available, please describe briefly the ethical, legal, or contractual restriction that prevents you from sharing it. Please also include an appropriate contact (web or email address) for inquiries (again, this cannot be a study author).*

*CODE AVAILABILITY*

*Please add a sentence to your data availability statement regarding the code used in the study, e.g. "The code used in the analysis is available in the Supporting Information files." and add the file accordingly.*

We have provided the code as supporting information file.

*ABSTRACT*

*ll.52ff: Please report statistical information as follows to improve clarity for the reader "22% (95% CI [13%,28%]; p</=). For example, line 52-53: "44/855 (5%) were high-risk of whom 43% (19/44, 95% confidence interval [29.7%, 57.8%]) had CIN2+." When reporting 95% CIs please separate upper and lower bounds with commas instead of hyphens as the latter can be confused with reporting of negative values.*

Revised.

*AUTHOR SUMMARY*

*1) l.70: Please change to "endocervical samples".*

We have revised to “cervical samples” – clinicians aim to collect ectocervical, endocervical and transformation-zone cells in their sample.

*2) l.72: Please temper claims of primacy of results by stating, "to our knowledge" or something similar or remove the word "novel".*

We have removed the word “novel”.

*INTRODUCTION*

*l.114: Please define 'UK' at first use.*

Revised.

*METHODS AND RESULTS*

*1) l.198: Please define 'hrHPV' at first use. Once you have introduced the abbreviation, please use the abbreviation throughout the manuscript. We have noticed that you alternate between using the abbreviation and "high-risk HPV".*

Revised.

*2) ll.311-314: Please revise the sentence. Do the three percentages refer to Ct values below 30, 30-35 and above 35?*

Revised.

*3) l.324: Please change to "showed".*

Revised.

*4) "A further 11% (=93.2%-81.8%)" - We are not sure that the calculation is very clear. We suggest writing "(=5 individuals; 41/44, 93.2%)".*

Revised as “A further 11% (5/44)”.

*5) l.346: Please change to "were".*

Revised.

*6) ll.349-351: Please revise for clarity.*

Revised.

*7) Figure 1: Please define 'hrHPV', 'CIN2', 'CIN3+'. Please note that the figure itself does not include the footnote 'a)'.*

Revised. We put the original footnote “a)” as legend.

*8) Figure 2: Please remove the definition "CIN2+, cervical intraepithelial neoplasia grade 2 or worse; CIN3+, cervical intraepithelial neoplasia grade 3 or worse" below the figure as you seem to be differentiating only between normal and abnormal cytology. Please explain in the figure description what 'Cyt+' and 'Cyt-' refer to. We also suggest adding an explanation that beta-globin serves as an internal control for sample cellularity and which HPV types are included. Please remember that all figures and/or tables should be self-explanatory on a stand-alone basis.*

Revised.

*9) Table 1: Please add a unit for age. Please note that footnote 'b)' is marked 'y' in the table.*

Revised.

*10) Table 2: Please note that there are no footnotes in the table. Please add the risk group definitions below the table.*

Revised.

*11) Figure 3: Please change to "All samples" and "Risk of CIN2+ and HPV Ct value in all samples".*

Revised.

*DISCUSSION*

*ll.371-372: Please revise for clarity.*

Revised.

*REFERENCES*

*1) Please use the word "accessed" instead of "cited" when specifying the date of (e.g. [accessed: 10/04/2024]).*

Revised.

*2) Please ensure that journal name abbreviations match those found in the National Center for Biotechnology Information (NCBI) databases (*[*https://eur01.safelinks.protection.outlook.com/?url=http%3A%2F%2Fwww.ncbi.nlm.nih.gov%2Fnlmcatalog%2Fjournals&data=05%7C02%7Cjiayao.lei%40ki.se%7C56c300cb666044c4b52208dce1260193%7Cbff7eef1cf4b4f32be3da1dda043c05d%7C0%7C0%7C638632800573843330%7CUnknown%7CTWFpbGZsb3d8eyJWIjoiMC4wLjAwMDAiLCJQIjoiV2luMzIiLCJBTiI6Ik1haWwiLCJXVCI6Mn0%3D%7C0%7C%7C%7C&sdata=0m6YFCIcCs%2BuyZ6tmWjoAX2wawusghR3zTGN8R%2FVK0s%3D&reserved=0*](https://eur01.safelinks.protection.outlook.com/?url=http%3A%2F%2Fwww.ncbi.nlm.nih.gov%2Fnlmcatalog%2Fjournals&data=05%7C02%7Cjiayao.lei%40ki.se%7C56c300cb666044c4b52208dce1260193%7Cbff7eef1cf4b4f32be3da1dda043c05d%7C0%7C0%7C638632800573843330%7CUnknown%7CTWFpbGZsb3d8eyJWIjoiMC4wLjAwMDAiLCJQIjoiV2luMzIiLCJBTiI6Ik1haWwiLCJXVCI6Mn0%3D%7C0%7C%7C%7C&sdata=0m6YFCIcCs%2BuyZ6tmWjoAX2wawusghR3zTGN8R%2FVK0s%3D&reserved=0)*), and are appropriately formatted and capitalized. For example, "New England Journal of Medicine" in reference [5] should be "N Engl J Med".*

Revised.

*SUPPLEMENTARY MATERIAL*

*In the published article, supporting information files are accessed only through a hyperlink attached to the captions. For this reason, you must list captions at the end of your manuscript file. You may include a caption within the supporting information file itself, as long as that caption is also provided in the manuscript file. Do not submit a separate caption file.*

*When SI files are contained with a single file:*

*Please label the file as 'S1 Supporting Information'.*

*Please apply alphabetical labelling to each table and figure contained within the S1 file. For example, 'Fig A' to 'Fig Z' and 'Table A' to 'Table Z'.*

*Plain text does not need to be labelled and can just be given a title as necessary. For example, 'Statistical Analysis Plan'.*

*Please cite tables/figures as 'Fig A in S1 Supporting Information' and/or 'Table A in S1 Supporting Information', for example.*

*Please cite plain text as, 'Statistical Analysis Plan in S1 Supporting Information', for example.*

*When SI files are uploaded as separate files:*

*Please label tables as 'S1 Table' (so on) and figures as 'S1 Fig' (and so on).*

*Any additional documents (protocols/analysis plans etc.) can be labelled as 'S1 Protocol', for example. Please cite items as exactly as labelled.*

Revised and all items are cited as required.

*SOCIAL MEDIA*

*To help us extend the reach of your research, please provide any X (formerly known as Twitter) handle(s) that would be appropriate to tag, including your own, your co-authors', your institution, funder, or lab. Please enter in the submission form any handles you wish to be included when we post about this paper.*

Information provided in submission form.

*------------------------------------------------------------*

*Comments from Reviewers:*

*Reviewer #1: Comments have well been answered and adjusted*

*Reviewer #2: Thanks to the authors for the clear and cogent responses to my feedback and associated changes to the manuscript. In most cases I agree and consider the items closed (assume this is the case unless otherwise indicated), but I have identified some follow-up points for further consideration that I have detailed below. In several cases the response to my question is clear but has not yet been reflected in the manuscript. In a small number of cases I have provided further suggestions.*

*Before providing the detailed follow-up points, I did want to make one general observation which is easy to fix and, on reflection, caused me quite a bit of confusion. When I read "risk" or "risk score", I immediately think of a continuous risk percentage between zero and one. Conversely, "risk category" or "risk strata" refer to ordered categories of risk. Given you are presenting both risk percentages (CIN2+) and risk strata (High/Medium/Low), I suggest choosing your preferred naming and ensuring it is used consistently throughout the manuscript text and tables/figures. This is of particular importance for more methodologically inclined readers because, as you know, choice of statistical model and approach hinges on the outcome variable type.*

Thank you for this suggestion. We have used “risk” to refer to a probability of proportion and “risk group” to refer to the ordered categories of risk throughout the paper.

*ADDITIONAL FOLLOW-UP POINTS*

*"We are following Castle and Katki (Nat Rev Clin Oncol 2016) who advocate 'equal management of equal risks' as a framework for developing screening guidelines and practice. It is precisely our different perspective that distinguishes this paper from the existing literature which focuses on sensitivity and specificity."*

*These sentences in the authors' response address my concern about choice and justification of methodology. I suggest this is reflected in the Introduction, including citing the work by Castle and Katki identified by the authors.*

Revised.

*Point 4.3.3.[a] The authors' response is very clear and helpful and, I believe, essential to any reader wishing to understand or replicate the analyses. As such, I would suggest this level of detail is included in the manuscript or (if preferred/required due to word count), in the supplementary materials.*

We have include more details in the methods and provided the dataset with codebook and Stata code used for the main analyses to allow readers to replicate the analyses.

*Point 4.3.3.[b] Further to the author's point "Note that for the primary model, there is no need for logistic regression, we can simply look at the observed proportions with CIN2+ in each group and calculated 95% confidence intervals using Wilson's method." I understand the primary analyses (as reflected in Table 1) and see how they are used in the definition of the three risk strata. That said, I am still unsure why the authors used this complicated combination of strata and descriptive univariate calculations when four multivariable models (one for each of the four binary endpoints represented by columns in Table 1) or, for that matter, a single model with an ordinal outcome, would have been simpler and allowed for assessment of relative salience of the various factors i.e., age group, HPV type, Ct value, and interactions thereof. That said, given the small number of patients and purpose of these analyses (informing the development of the risk strata as opposed to definitively establishing risk percentages), I accept the current analyses are sufficient.*

Thank you.

*Point 4.4.2. I am not convinced you have addressed my point here. In the results you still refer to "PPV" (lines 348 and 357) and it is not included in your methods; either as a calculation using these data or as a reference point sourced from prior work. Relatedly, I really am struggling to understand what you are trying to communicate here given your response to point 4.4.2 suggests you believe PPV is not useful in and of itself as a comparator, but you continue to use it in this way in the manuscript.*

Apologies for the confusion. We have removed “PPV” throughout the paper and used “percentage” or

“proportion”.

*6.1.1 Perhaps I am missing it, but "hrHPV" does not yet appear to be defined in the manuscript text.*

Revised.

*6.2.1 "Although values over 32.5 are outliers on the box and whiskers plots, they were all within the manufacturer's adequate range." Suggest including this (or similar) in your discussion given you present the results.*

Revised.

*6.3.1 The changes read very well.*

Thank you.

*6.3.2 This is OK, but I still have a concern about the use of the phrase "clear gradient" given gradient means gradual change and you only have three levels.*

Thank you. We have replaced “gradient” with “trend”.

*6.3.3 & 6.4.3 These responses are excellent. Well done.*

Thank you.

*6.4.4 & 7.3 Constraining your results to informing future pilot implementation programs and screening studies (as opposed to definitively establishing risk of outcomes or immediately changing current clinical practice) addresses my concerns here and elsewhere.*

Thank you.

*Figure 2 notes include definitions for CIN2+ and CIN3+ but these terms are not included in the title or figure itself.*

Revised.

*Any attachments provided with reviews can be seen via the following link:*

*------------------------------------------------------------*

*General Editorial Requests*

*1) We ask every co-author listed on the manuscript to fill in a contributing author statement. If any of the co-authors have not filled in the statement, we will remind them to do so when the paper is revised. If all statements are not completed in a timely fashion this could hold up the re-review process. Should there be a problem getting one of your co-authors to fill in a statement we will be in contact. YOU MUST NOT ADD OR REMOVE AUTHORS UNLESS YOU HAVE ALERTED THE EDITOR HANDLING THE MANUSCRIPT TO THE CHANGE AND THEY SPECIFICALLY HAVE AGREED TO IT.*

I believe that all authors have declared all competing interests. Please let us know if you are missing any.

*2) Please ensure that the paper adheres to the PLOS Data Availability Policy (see* [*https://eur01.safelinks.protection.outlook.com/?url=http%3A%2F%2Fjournals.plos.org%2Fplosmedicine%2Fs%2Fdata-availability&data=05%7C02%7Cjiayao.lei%40ki.se%7C56c300cb666044c4b52208dce1260193%7Cbff7eef1cf4b4f32be3da1dda043c05d%7C0%7C0%7C638632800573855594%7CUnknown%7CTWFpbGZsb3d8eyJWIjoiMC4wLjAwMDAiLCJQIjoiV2luMzIiLCJBTiI6Ik1haWwiLCJXVCI6Mn0%3D%7C0%7C%7C%7C&sdata=yofrVQlln0kMhGiLd9yOVuLXPABs0L66EQ7H1GqyYHo%3D&reserved=0*](https://eur01.safelinks.protection.outlook.com/?url=http%3A%2F%2Fjournals.plos.org%2Fplosmedicine%2Fs%2Fdata-availability&data=05%7C02%7Cjiayao.lei%40ki.se%7C56c300cb666044c4b52208dce1260193%7Cbff7eef1cf4b4f32be3da1dda043c05d%7C0%7C0%7C638632800573855594%7CUnknown%7CTWFpbGZsb3d8eyJWIjoiMC4wLjAwMDAiLCJQIjoiV2luMzIiLCJBTiI6Ik1haWwiLCJXVCI6Mn0%3D%7C0%7C%7C%7C&sdata=yofrVQlln0kMhGiLd9yOVuLXPABs0L66EQ7H1GqyYHo%3D&reserved=0)*), which requires that all data underlying the study's findings be provided in a repository or as Supporting Information. For data residing with a third party, authors are required to provide instructions with contact information for obtaining the data. PLOS journals do not allow statements supported by "data not shown" or "unpublished results." For such statements, authors must provide supporting data or cite public sources that include it.*

We have provided the data as supporting information and revised the statement.

*3) Please review your reference list to ensure that it is complete and correct. If you have cited papers that have been retracted, please include the rationale for doing so in the manuscript text, or remove these references and replace them with relevant current references. Any changes to the reference list should be mentioned in the rebuttal letter that accompanies your revised manuscript.*

Done.
